# Supplementary material for: Schizophrenia-related cognitive dysfunction in the Cyclin-D2 knockout mouse model of ventral hippocampal hyperactivity
Source: Transl Psychiatry. 2018 Oct 9;8:212. doi: 10.1038/s41398-018-0268-6 (PMC6178344; doi:10.1038/s41398-018-0268-6)
Supplement: Supplementary file 1 — Supplementary Information [file 41398_2018_268_MOESM1_ESM.docx]

Supplementary Information

**Schizophrenia-related cognitive dysfunction in the Cyclin-D2 knockout mouse model of ventral hippocampal hyperactivity**

***Running title:*** *Modelling cognitive impairments in the prodromal state*

**Christina M. Grimm^1^, Sonat Aksamaz^1,*^, Stefanie Schulz^1,*^, Jasper Teutsch^1^, Piotr Sicinski^2^, Birgit Liss^1^, Dennis Kätzel^1,#^**

^1^ Institute for Applied Physiology, Ulm University, Germany

^2^ Dana-Farber Cancer Institute and Harvard Medical School, Boston, MA, USA

* These authors contributed equally to this work.

^#^ Correspondence: dennis.kaetzel@uni-ulm.de, Tel. +49 731 500 33770, Institute for Applied Physiology, Ulm University, N26, Albert-Einstein-Allee 11, Germany

# Supplementary Methods

## Animals

All experiments were approved by the Regierungspräsidium Tübingen, Germany. Cyclin D2 knockouts (*Ccnd2^−/−^* or CD2-KO, C57BL/6J background) had been generated before via homologous recombination disrupting exons 1 and 2 of *Ccnd2* ^1^ and were provided by Peter Sicinski’s laboratory (Harvard Medical School, USA). CD2-KO mice and their littermate controls were bred from heterozygous parents at the animal facility of Ulm University. Mice were genotyped from ear notches using primers 5’-GCTGGCCTCCAATTCTAATC-3’, 5’-CCAGATTTCAGCTGCTTCTG-3’ and 5’- CTAGTGAGACGTGCTACTTC-3’. Experimental animals were housed on a 13/11-hour light/dark cycle in a temperature and humidity controlled holding room at the institute of Applied Physiology of Ulm University in immediate vicinity to behavioural testing rooms. Their individually-ventilated cages (IVC) contained sawdust, sizzle nest and one cardboard house (Datesand, UK) as enrichment. Except for final experiments requiring isolation for 6 days (sucrose preference and nest building), animals were always group-housed. Heterozygous littermates (CD2^+/-^) were not included in experimental procedures, but were sometimes kept as cagemates to avoid single-housing.

Food was available ad libitum during the assessment of tasks driven by spontaneous or drug-induced behaviour. For rewarded tasks, mice were put on a food deprivation schedule and kept at 85-95 % of individually determined average free-feeding weights. Water was provided ad libitum at all times. All experimental procedures were performed during the light phase, except for assessment of sucrose preference and nest building. Except for the dose-response curves in separate wildtype cohorts, the challenge conditions in the 5-choice serial reaction time task, and the cognitive flexibility assays, all behavioural experiments were conducted in both sexes.

## Behavioral experiments

### Novelty-induced locomotor activity

For measurement of locomotor activity, mice were put into a novel clear plastic cage (425 x 266 x 185 mm; Eurostandard Typ III, Tecniplast, G) filled with clean sawdust and left to explore for 2 hours. CCTV cameras (Sentient, UK) installed centrally above the open-field cage were used to monitor each animal. Video-recordings from 8 cage-stations were assembled into a single image frame through a CCTV-system (Dahua Inc, China), digitized through an A/D converter (TheImagingSource, G), and fed into ANY-maze (San Diego Instruments, USA), for video-tracking of movement. The total distance travelled, the number of rotations and the average distance to the border of the arena were extracted from ANY-maze in either 5-minute intervals or for the whole time of the experiment. The number of rotations of the animal’s body is determined as specified in http://www.anymaze.co.uk/a-detailed-description-of-the-any-maze-measures.pdf. Rotations are defined as 360 degree turns, that are completed without full reversal of the animal’s path. Rotations in either direction are counted and summed up for this measure.

### Pharmacological experiments during locomotion in the open field

Pharmacological dose-response experiments were performed in the same experimental setup. To assess the effects of the D1-antagonist SCH23390, the D2-antagonist raclopride and the mGlu2/3R-agonist LY379268 on novelty-induced locomotion in CD2-KO mice, each drug was injected i.p. at an injection volume of 10 ml/kg prior to a 1 h exposure in the novel open-field. S(-)Raclopride-(+)tartrate salt (Sigma, G) was diluted in 0.3 % TWEEN80/saline and injected 60 min before the start of the run. SCH23390 hydrochloride and LY379268 disodium salt (Tocris, G) were diluted in saline and injected 30 min or 45 min, respectively, before the run.

For dose-response assessment of these drugs, separate adult male C57BL/6 cohorts were used in a between-subject design (4-9 mice per sub-group); either raclopride, SCH23390 or LY379268 were injected 30 min, 30 min, or 45 min, respectively, before a 30 min exposure to the novel open-field, during which the efficacy against novelty-induced locomotor activity was assessed. Immediately after this 30 min run, 0.2 mg/kg MK-801 maleate (in saline) was injected and the animals were returned to their locomotor cages for another hour to assess the efficacy of each drug / dose against locomotion induced by NMDA-receptor blockade.

Latin-square designs with a fixed order of doses, counterbalanced for starting dose and genotype, were used for all within-subject studies. The order was chosen so that the next-lower dose would be used on the subsequent test, except for the vehicle-session, which was succeeded by a session with the highest dose.

In contrast to the other drugs, amphetamine (D-amphetamine-hemisulfate diluted in saline) was applied only in a between-subject design, using doses that were based on prior experience ^2^. Also, animals were exposed to the novel locomotor boxes for a 30 min pre-run before i.p. injection of amphetamine (10 ml/kg for vehicle and 2 mg/kg and 5 ml/kg for the 1 mg/kg dose), which was immediately followed by a further 1 h run in the same locomotor box, that each animal had been in before.

Drug doses for experiments in CD2-KO mice were chosen based on the prior dose-response curves conducted in wildtype mice (see Figure 1), aiming to pick doses that were effective in reducing novelty- and MK-801-induced locomotion, but not strongly sedative. The dose-ranges tested in those pilot experiments were estimated from the literature for LY379268 ^3^ and SCH23390 ^4,5^, and from our own data ^6^ for amphetamine, raclopride, and MK-801.

### Spatial rule-shift and rule-reversal learning

Spatial reference, rule-shift, and reversal learning were assessed in male CD2 knockouts and respective control animals under food deprivation (85-90 % of free-feeding weight) with water available *ad libitum*. Prior to testing, mice were habituated to the transparent, plus-shaped maze, and were free to consume the reward (condensed milk, diluted 1:1 in drinking water) from the metal food wells at the ends of each goal arm (East and West). An additional extra-maze cue in form of a Duplo^TM^ tower was placed next to the north-western corner of the maze. Each mouse was assigned a goal arm or rule counter-balanced within each genotype, and a pseudo-randomized start arm (North or South) was assigned for every trial. 180° rotation of the maze and cleaning with 50 % ethanol were performed regularly to abolish potential intra-maze cues to be associated with the reward. The criterion to be reached in each paradigm was set to 85 % (17/20) correct choices in four consecutive training blocks of 5 trials each. Once met, the location-reward association was changed and mice were trained on the next paradigm (spatial rule-shift) until the same criterion was fulfilled, after which they were trained on the third paradigm assessing spatial reversal learning. Before the alteration of the rule, mice were given 2 blocks of olfactory-control trials, in which the reward was given only after a mouse had made its choice, to exclude the potential confound that mice could determine the correct choice by smelling the reward.

The initial training paradigm of spatial reference learning followed an ego-centric rule (e.g. no matter where mice started, they always had to turn left to obtain a reward). After this, spatial rule-shift learning was implemented by training mice according to an allo-centric rule (e.g. no matter where mice started, they always had to turn into the Eastern arm of the maze). Finally, spatial rule-reversal learning was done by reversing the association between arm and reward, but remaining within the allo-centric framework (e.g. a mouse that needed to go to the Eastern arm before, now needed to go to the Western arm).

For analysis of rule-shift and rule-reversal learning, two primary measures were used: the number of blocks (of 5 trials) needed to reach the stated learning criterion using a *t*-test, and a repeated-measures ANOVA across the last 50 % of the blocks that were needed on average to reach criterion, rounded up to the next even number. The latter measure was defined as such, because some tasks were acquired faster than others. We deem the second half of blocks more relevant to the underlying question of cognitive flexibility, because at the beginning the new rule is puzzling for every mouse, including controls, so that a difference between genotypes is not expected in the first blocks. Nevertheless, an analysis across all blocks needed to reach criterion on average is also provided in Supplementary Table 3a, which also lists the number of blocks needed on average to reach criterion for each test.

### Spatial novelty preference

Spatial novelty preference, an indicator of spatial short-term habituation, was tested in a Y-shaped maze with three transparent arms at a 120° angle from each other as previously described ^2,7^. The maze floor was covered with clean sawdust, into which dirty bedding from unfamiliar mice of the same sex was introduced to enhance exploratory behaviour. Each mouse was put into a separate holding cage (placed close to the Y-maze) for 8-10 minutes before testing began. In the sample phase (5 min) animals could explore the start arm and one of the goal arms, while the second goal arm (whose location was counterbalanced between groups), was closed by a non-transparent door. Subsequently animals were removed into the holding cage for a 1 min delay phase, in which the door was removed from the other goal arm, and the sawdust of the entire maze was mixed. Subsequently, mice were transferred back to the maze for a 2 min test phase. Location was video-tracked through ANY-maze (San Diego Instruments, USA). Novelty preference was calculated as time spent in the novel goal arm divided by time spent in both goal arms combined. As a secondary measure, a preference score was calculated based on the number of entries into the novel goal arm divided by the number of entries into both goal arms.

### Novel-object recognition

Mice were habituated to a dark grey open field (40 x 40 cm, 25 cm wall height) 5 times for 5 min over 2 days prior to testing and one last time (5 min) on the morning of the testing day, before the first animal of the cohort was tested. On the test day mice were exposed to two identical copies of object X for 10 minutes. Mice were then removed from the open field for 2 min, during which the initial objects were replaced by an identical copy of object X and a novel object Y. Mice were then reinserted into the open field and left to explore for 5 min ^2,8^. Direct exploration of the objects was manually scored on-line by the experimenter blinded for genotype, and videos were recorded using ANY-maze (San Diego Instruments). Objects and the arena were cleaned with 50 % ethanol and water between animals. The identity of the first object and its position in the arena were counterbalanced within each genotype. The preference for the novel object was calculated as the ratio of the time spent with the novel object divided by the time spent with both objects combined. As a secondary measure of preference the ratio of the number of contacts with the novel object divided by the number of contacts with both objects was used.

### Five-choice serial-reaction time task (5-CSRTT)

Testing was conducted as previously described ^2,9,10^ in 8 ventilated operant chambers (Med Associates, VT, USA) each containing one wall with five nose-poke holes fitted with a recessed stimulus light. An infrared beam transecting each hole was installed for detection of nose-pokes. A food magazine (receptacle) was placed opposite to the 5-hole array. Here, food reinforcement in form of strawberry milk (40 µl, Müller^TM^ Milch) was delivered. An additional break-beam-based detector for head entries into the receptacle was situated above the milk dispenser. A house light was located in the upper left corner of the wall that contained the food magazine. Operant chambers were connected to a Smart Control Panel and an interface to a computer allowed for data acquisition in the MED-PC software (Med Associates, USA). The different protocols for training and testing were implemented using the Med-PC programming language.

Male and female CD2 knockout and wildtype mice were kept on 85-90 % of free-feeding baseline weight measured over 3 days immediately prior to the start of food-deprivation. On the next days, they were habituated to the consumption of the reward in the operant chamber over 2-5 10 min sessions. Once they readily consumed the milk, they were trained on a habituation protocol for up to 20 sessions, in which all 5 holes of the wall were illuminated and mice were rewarded for poking into either one of the illuminated holes. The first trial was initiated via a rewarded head entry into the receptacle, and all subsequent trials were initiated after a waiting time (inter-trial interval, ITI) of 2 s after the start of the reward collection (receptacle entry). Mice were maintained on habituation training for at least 17 and at maximum 21 sessions, and they were admitted to the subsequent training on the 5-CSRTT once they met the criterion of at least 30 correct responses in three consecutive sessions.

Subsequently, mice were trained on the 5-choice training protocol over 4 stages of increasing difficulty, as determined by the length of time during which the stimulus light was switched on (stimulus duration, *SD*) and the length of the waiting time (inter-trial-interval, *ITI*) between reward collection from the previous trial and presentation of stimulus lights for the next trial. The 5-CSRTT built on the basic operant cycle that mice acquired during the habituation training, but with various adaptations: only one, pseudo-randomly selected, hole of the 5-hole array was lit after the *ITI*. The animal had a limited hold time (*LH*), i.e. an allowed response time, that exceeded the presentation of the stimulus light by 2-10 s depending on the stage - correct pokes within this time would be rewarded. Failure to respond at all during the time defined by the LH, was counted as an *omission* and was punished by omission of reward and a 5 s time-out (house light switched off) before the next trial started. Equally, responses *before* the actual illumination of any hole were counted as *premature responses*, and pokes into holes that were not illuminated (during the response time (LH)) were counted as *incorrect responses*. Premature and incorrect responses were punished in the same way as omissions. Only poking into the illuminated hole within the response time was counted as a *correct response,* resulted in immediate stopping of the hole-illumination and was rewarded. Repeated poking into the correct hole (after it was no longer illuminated) was counted as *perseverative response,* but was not punished.

Relative measures that were used as performance readouts were calculated as follows (whereby: C, correct response; I, incorrect responses; O, omissions; Pre, premature responses; Prs, perseverative responses):

% accuracy = 100 * #C/(#C + #I)

% correct = 100 * #C/(#C + #I + #O)

% omissions = 100 * #O/(#C + #I + #O)

% premature = 100 * #Pre/(#C + #I + #Pre) = 100 * #Pre/(total number of trials)

% perseverative = 100 * #Prs/( #C + #I + #Pre + #Prs + responses during time-outs)

= 100 * #Prs/(total number of nosepokes)

The stages and challenge protocols were characterized by the following parameters:

|  | **Parameters of training** | | | **Criteria to transition (on 2 consecutive days)** | | | |
| --- | --- | --- | --- | --- | --- | --- | --- |
| **Stage** | **SD (s)** | **LH (s)** | **ITI (s)** | **# correct** | **% correct** | **% accuracy** | **% omissions** |
| 1 | 20 | 30 | 2 | >= 30 | >= 30 | - | - |
| 2 | 10 | 30 | 2 | >= 50 | >= 50 | - | - |
| 3 | 8 | 20 | 5 | >= 50 | >= 50 | - | - |
| 4 (baseline) | 4 | 7 | 5 | >= 50 | - | >= 80 | <= 50 |

Upon reaching the criterion for transitioning from stage 4, mice were trained for at least one additional day in that stage. Subsequently, mice were subjected to four challenge paradigms in the stated order to assess attention and impulsivity. Testing on the challenge protocols was performed on two consecutive days, with at least one baseline training (stage 4) between different challenges. The challenges were characterized by the following parameters:

| **Challenge** | **SD (s)** | **LH (s)** | **ITI (s)** | **Distraction (s)** |
| --- | --- | --- | --- | --- |
| SD reduction (attention challenge) | 1 | 7 | 5 | None |
| ITI increase (impulsivity challenge) | 2 | 7 | 9 | None |
| Distraction: 0.5 s | 2 | 7 | 5 | 0.5s |
| SD reduction & Distraction 1 s | 1 | 7 | 5 | 1s |

The distraction refers to the time for which the house light is switched off once at a random time during the ITI (but not in the first or last second of the ITI). The SD and LH were reduced only mildly compared to the baseline protocol (4 s to 2 s) to enforce the tight focus on the 5-choice wall.

For analysis, the performance parameters from the two days of each challenge were averaged and compared to the performance in the baseline session immediately preceding the given challenge. Mice that did not reach the transition criterion on stage 4 by the 60^th^ training session were not tested in the subsequent challenge protocols; since many females dropped out by that criterion, behaviour under challenges was only analysed in male mice.

### Operant rule shift learning

Male CD2 knockout and wildtype mice were subjected to operant rule-shift learning after the 5-CSRTT. Mice were at first trained on a simplified 5-CSRTT task protocol, where only holes 2 or 4 of the 5-hole wall were illuminated for 10 s (SD = 10 s, LH = 12 s, ITI = 2 s) and poking into the illuminated hole was rewarded by 40 μl strawberry milk. Mice were trained, until the criterion of 80 % accuracy on two consecutive training days was reached. For rule-shift learning, the reinforcement contingencies were changed such that the illumination of a hole - although continued in the same fashion as before - no longer indicated if poking into it was rewarded. Instead, the spatial position now indicated the correct choice, i.e. either hole 2 or 4 were rewarded (depending on the individual mouse, assignment counterbalanced within genotypes), no matter if it was illuminated or not. Here, the primary measure for successful rule-shift learning was percent accuracy for the choice of the unlit over the lit hole, in trials where the rewarded hole was unlit (the accuracy in trials were the rewarded hole was also lit, i.e. the old and the new rule applied simultaneously, was virtually 100 % in every mouse, therefore these trials were not included in the analysis). The two types of trials were pseudo-randomized retaining a 50:50 probability. Temporal parameters were identical to those used in the previous learning: SD = 10 s, LH = 12 s, ITI = 2 s.

### Rewarded alternation on the T-maze

Rewarded alternation was assessed as previously described ^2,11^, but in a T-shaped maze with a red PVC floor, transparent Perspex walls, and metal food wells at the end of each goal arm (all arms: w 10 cm, l 40 cm; wall-height 10 cm). Testing was conducted in food-deprived mice, kept at 85-90% of their free-feeding weight. Animals were habituated to the maze and the condensed milk reward prior to testing - at first in groups (all mice from each home cage), then individually - to ensure that mice were moving on the maze and consuming the reward without anxiety. For training and testing, 10 trials per day were performed and each trial consisted of a sample and a choice run. In the sample run mice were placed in the start arm facing the experimenter and forced into one of the goal arms to gain a reward. The forced direction was randomly assigned, with no more than three consecutive trials in the same direction, and overall equal numbers of left and right allocations. Mice were then removed from the maze for a defined intra-trial interval or “delay” (1 s, 5 s, 20 s or 60 s, as indicated), during which the non-transparent door from the second goal arm was removed. For the choice run, mice were returned to the maze start arm facing the maze, and allowed to make a free choice between both goal arms, of which the previously blocked (unvisited) one was rewarded. A decision was counted when the mouse had crossed the T-junction and all four paws were within a goal arm. Mice received 5 days of training with 10 trials each and an inter-trial interval of 3-5 minutes (round robin or “blocked” design). Sample and choice runs during training were separated by an intra-trial interval (delay) of 5 s. After training, mice were tested with different intra- and inter-trial intervals (as indicated in the corresponding figure) to manipulate the difficulty of the task and to replicate conditions used in related previous studies ^2,12^. Each of these challenge protocols was conducted for 2 consecutive days and the performance of both days was averaged for analysis. For the protocol with 1 s delay (i.e. the choice trial started immediately after the mice were removed from the food well in the goal arm that they had visited in the sample phase), a massed design was used, whereby all 10 trials were performed consecutively by each animal, with an inter-trial-interval of 20-25 s between them. For all other challenge protocols a blocked design was used with an inter-trial-interval of at least 3 min.

For pharmacological assessment of the effect of LY379268 on T-maze performance, mice were trained again in the baseline protocol (blocked design, 5 s delay) for 6 days, before a schedule of 3 test days interleaved with 2-3 gap days started. On the test days, LY379268 (1 or 3 mg/kg) or vehicle was injected 50-60 min before the start of testing, and animals were given 10 trials in a massed design with 1 s delay (the protocol which showed, qualitatively, the largest impairment in CD2-KO mice in both sexes). On the 1-2 gap days that followed the first 2 test days, no training was conducted, while on the day before the next test day animals were given 10 trails in the baseline protocol (blocked design, 5 s delay) for re-training. The doses each animal received were determined by a latin-square design whereby the starting dose was counterbalanced within each genotype.

### Elevated plus maze

Unconditioned anxiety was assessed on a plus-shaped maze consisting of two closed and two open area arms made entirely out of grey PVC plastic, both oriented opposite to each other and elevated 72 cm above the ground. Following habituation to a novel holding cage in the testing room for 5-7 min, male and female CD2 knockout and wildtype mice were placed at the junction of the four arms of the maze, facing one of the open arms, and left to explore for 5 min, during which they were video-tracked by ANY-maze. Entries to each zone were defined by the position of the animal’s body centre. A preference score was calculated as the time spent in the open arms divided by the time spent in the closed arms (excluding the centre) as the primary, inverse measure of anxiety.

### Sucrose preference

Mice were single-housed on the first day of testing. Testing was conducted during the dark phase over five consecutive nights, while animals were kept in their cages (single-housed) with just the water bottle during the day (ca. 9 a.m. – 5 p.m.). On the first night, mice were habituated to being single-housed and to the presence of two water bottles, placed 10 cm apart on the same side of the IVC home cage. Following this habituation night, mice were given the choice of drinking either plain water or 1 % w/v sucrose/water during nights 2 and 3. During nights 4 and 5, 10 % sucrose solution was provided instead of 1 %. Sucrose solution containing bottles were removed the following morning, leaving only the option to drink water during the day. Intake of water and sucrose solution over-night was measured daily by weighing the bottles in the evening and morning. The preference for sucrose was calculated as percentage volume of sucrose consumption over total volume of liquid taken in. Positioning of sucrose solution bottle was counterbalanced for genotype and kept the same throughout testing for each animal.

### Nest building

Assessment of nest building was performed overnight as previously described ^13^, immediately after the last night of sucrose-preference assessment. Mice were single housed in a cage containing sawdust and 2.3-2.5 g Nestlet^TM^ material. All enrichment items were removed beforehand and food pellets were provided in the saw dust instead of the food hopper. Unused Nestlet^TM^ material was weighed the next morning and the quality of the nest was rated on a 5-point scale by two observers blind to genotype according to established scoring guidelines ^13^. Intermediate scores were assigned in cases of discrepancy between weight of unused Nestlet^TM^ material and overall appearance of the nest.

### Reciprocal social interaction

Reciprocal social interaction was assessed in fresh cages of the same sizes as the locomotor cages, but with darker walls (Typ III made from H-Temp Polysulfone). CD2-KO or wildtype control mice were left to interact freely for at least 16 min with unfamiliar stimulus mice, that were of the same stain and gender, but several months younger in age (although still adult). Stimulus mice were inserted into the open-field 2-5 min before the test mice were added. Social interactions such as sniffing, taxing and grooming, undertaken by the test mice, was scored by an observer blind to genotype, while aggression or sexual behaviour was not counted. One male was excluded from the analysis due to repeated aggressive behaviour, which was otherwise not observed in our cohort during this test.

## Statistical analysis

Normality of data was assumed except for the nest building parameters. Repeated-measures ANOVA was used for within-subject subject data, usually followed by univariate ANOVA to compare between genotypes at each data point. Between-subject data was assessed with univarate ANOVAs (indicated as “ANOVA” throughout the main text), partially followed by pairwise *t*-tests conducted in the two gender groups separately, were applicable (see Supplementary table 1). In cases were normal data distribution could not be assumed (nest-building), the Mann-Whitney U Test was applied as non-parametric tests. Results are reported as “significant” if *P* < 0.05 and as “not significant” (n.s.) if P ≥ 0.05. Trends (0.5 ≤ P < 0.1) are reported individually, especially were *n* numbers were lower. All statistical analysis was performed with SPSS version 24 (IBM, US).

# Supplementary Figures


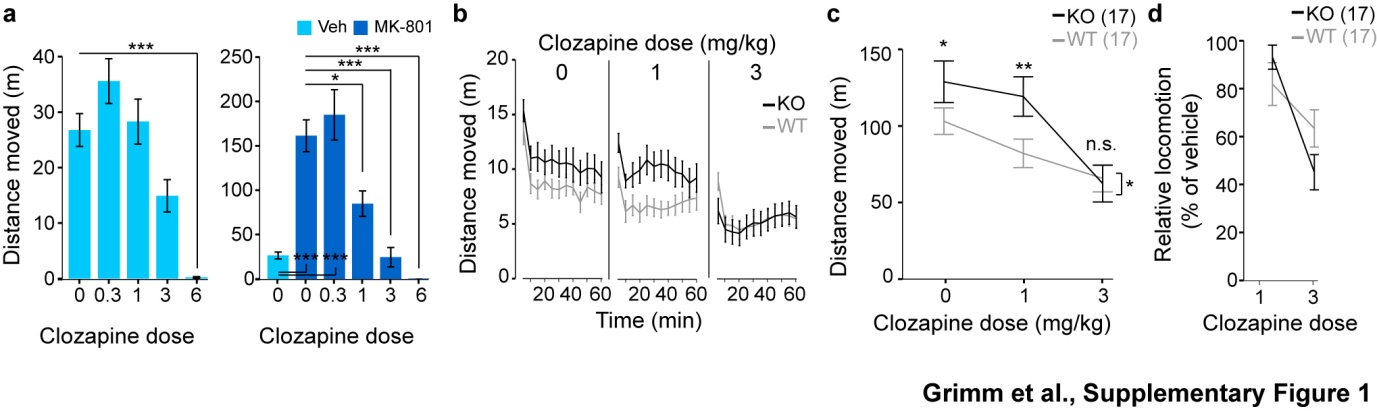


**Supplementary Figure 1. The effect of clozapine on novelty-induced hyperlocomotion in CD2-KO mice.** All experiments and analysis are analogous to those presented for raclopride, SCH23390 and LY379268 in main Figure 1. (**a**) Dose-response measurements of the efficacy of clozapine in reducing novelty-induced (left) or MK-801-induced (right, dark-blue indicates injections of 0.2 mg/kg MK-801, light-blue is vehicle) hyperlocomotion using the stated doses (x-axes). A separate cohort of adult male C57BL6/J wildtype mice was used in a between-subject design with 4-7 mice per subgroup (dose). Clozapine was dissolved in a vehicle of 10 % DMSO/saline and injected i.p. at 10 μl/g ca. 15 min before the start of the pre-run of 30 min assessing novelty-induced locomotion (left). Immediately afterwards, 0.2 mg/kg MK-801 was injected and locomotor activity was assessed for a further 60 min in the same cage. (**b**) Within-subject assessment of clozapine at the stated doses on novelty-induced locomotion in male and female CD2-KO mice and controls, plotting distance moved in 5 min intervals. Clozapine was injected i.p. 30 min prior to the start of the locomotor activity test. (**c**) The same data and within-subject assessment as in (b), but plotting total distance moved during the test phase of 1 h. (**d**) Same data as in (c), but plotting distance moved in 1 h (as %) relative to the vehicle (0) value in each animal. Wildtype controls, grey; CD2-KO, black; numbers in brackets denote *n* for each group. All doses stated in mg/kg. * *P* < 0.05, ** *P* < 0.01, *** *P* ≤ 0.001. Significance stars in (**a**) represent between-subject comparisons with a Tukey-HSD post-hoc test following after a significant effect of dose in a one-way ANOVA (*P* < 0.0005 in both cases). Significance stars in (**c**) and (**d**) indicate effects of genotype identified at a single dose (above data points of each dose, univariate ANOVA), or across all doses (vertical bracket at the side, repeated-measures ANOVA). Error bars, S.E.M..

Note that a dose of 1 mg/kg clozapine, which is effective in reducing MK-801-induced locomotion, did not normalize novelty-induced hyperlocomotion in CD2-KO mice, while a dose of 3 mg/kg normalized both types of hyperlocomotion. 1 mg/kg clozapine reduced novelty-induced locomotion within controls (*P* = 0.027, paired t-test) but not within CD2-KO mice (*P* = 0.104). See Supplementary Table 1d for further statistical analysis of this data.


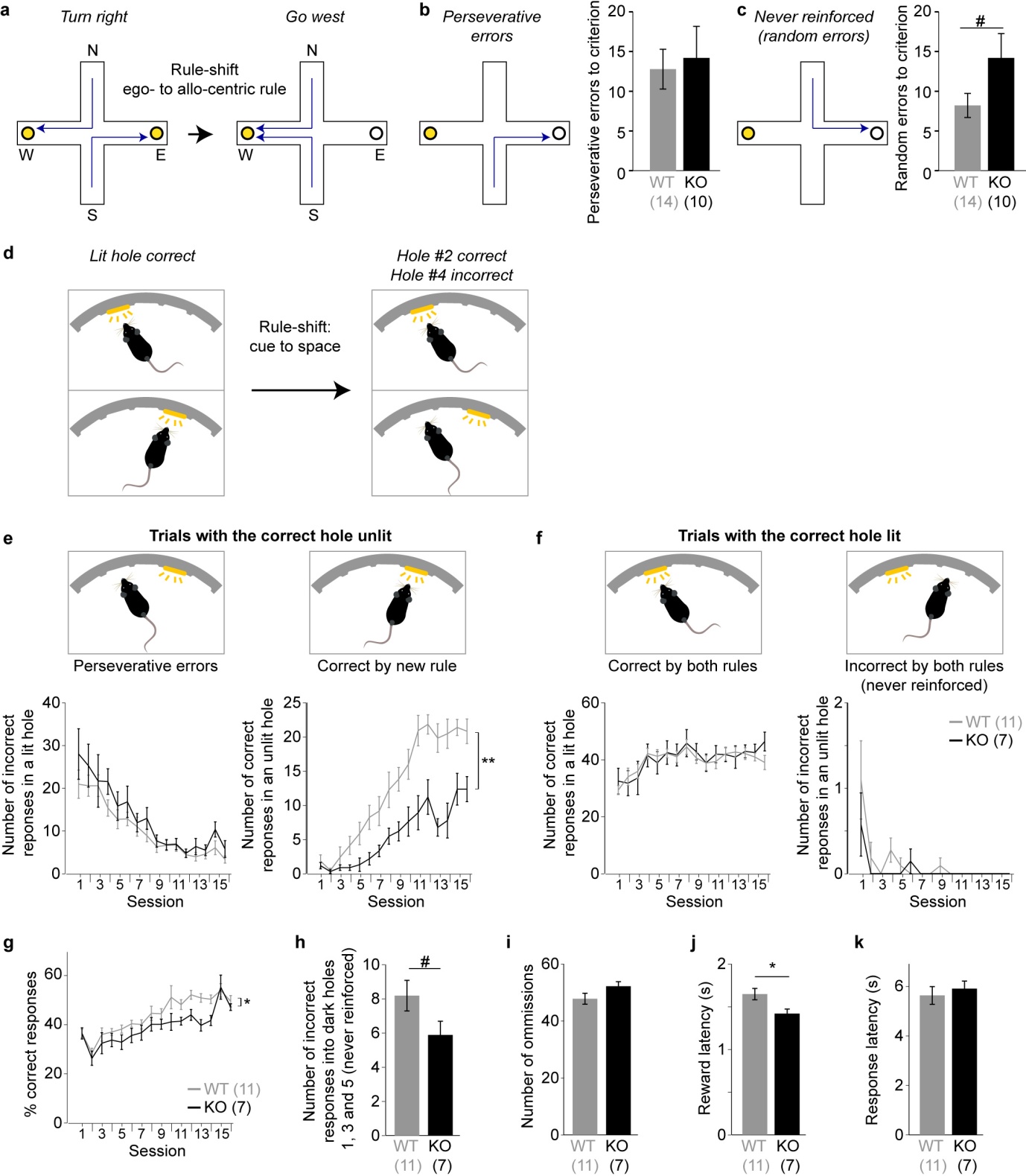


**Supplementary Figure 2. Analysis of error types on rule-shift tasks.** (**a-c**) Spatial rule-shift learning in the plus-maze. (**a**) Illustration of the task displaying one out of two possible rule-sets for each phase (see Supplementary Methods and main Figure 2). (**b**) Illustration (left) and number (right) of perseverative errors. (**c**) Illustration (left) and number (right) of choices that represent errors according to both the old and the new rule (“random” or “never-reinforced” errors). (**d-k**) Rule-shift learning in the operant box (see Supplementary Methods and main Figure 2). (**d**) Illustration of the task displaying one out of two possible rules during the shift-phase (right). (**e**) Illustration (top) and absolute numbers (bottom) of incorrect (left) and correct (right) responses in those trials where the incorrect choice hole was illuminated while the correct choice hole was unlit. Responses into the lit hole (left) represent perseverative errors, as they would be correct according to the rule reinforced in the prior learning phase. Responses into the unlit choice hole (right) represent correct responses according to the new, but not the old, rule. The decrease of errors and increase of correct responses represent the rule-shift learning and determine the accuracy displayed in main Figure 2k. Note that mice of both genotypes show a similar decrease of perseverative errors, but knockouts show a significantly slower increase of correct responses. (**f**) Illustration (top) and absolute numbers (bottom) of correct (left) and incorrect (right) responses in those trials where the correct choice hole was also illuminated while the incorrect choice hole was unlit. Responses into the lit hole are correct by both the old and the new rule, and they are made to an equal extent by both genotypes. Responses into the unlit hole were never reinforced by either the old nor the new rule, and therefore represent random errors, which are virtually never made after the first session. (**g**) % correct responses across both types of trials are calculated as the ratio of the number of trials with correct responses and the total number of trials in a given session. The total number of trials is the sum of the following types of trials: correct responses according to new rule, incorrect responses due to perseverative or random (“never-reinforced”) errors, and omissions (absence of response within the limited-hold time of each trial of 12 s). The %correct measure reveals that CD2-knockouts display lower overall performance in terms of obtaining the maximum number of rewards per total number of trials. (**h**) Average number of responses into holes 1, 3 and 5, which were never illuminated nor reinforced by any rule or in any of the trials across the first 16 sessions. (**i**) Average number of omissions across the first 16 sessions. (**j**) Average reward latency across the first 16 sessions in seconds. Note that CD2-knockouts appeared to have a shorter reward latency, which indicates a higher incentive motivation. (**k**) Average response latency for correct responses across the first 16 sessions. Horizontal lines indicate effects of genotype determined by *t*-test, vertical brackets indicate effects of genotype determined by repeated-measures ANOVA. # *P* < 0.1, * *P* < 0.05, ** *P* < 0.01. WT, wildtype controls; KO, CD2-knockouts. See Supplementary Table 3 for further statistical analysis of this data.

# Supplementary Tables

**Supplementary Table 1a: Behavioural between-subject data analysed with univariate ANOVA.** 5CSRTT, 5-choice-serial-reaction-time task; Fig., figure panel containing the analysed data; Ctrl, littermate wildtype controls, KO, CD2-KO mice; df, degrees of freedom; n, number of animals in the indicated group; #, absolute number of observations of the stated parameter; F, F-value of the ANOVA; p, *P*-value, t, t-value of the *t*-test. Purple shading indicates adjustments of df and *P*-value due to unequal variances. Green shading indicates significant *P*-value (< 0.05), faint green shading indicates near-significant *P*-value (0.05 – 0.06).

**Supplementary Table 1b: Behavioural within-subject data analysed with repeated-measures ANOVA.** 5CSRTT, 5-choice-serial-reaction-time task; f, females; m, males; SD, stimulus duration in 5-CSRTT; ITI, inter-trial interval in 5CSRTT; Fig., figure panel containing the analysed data; Ctrl, littermate wildtype controls, KO, CD2-KO mice; df, degrees of freedom in the ANOVA; n, number of animals in the indicated group; #, absolute number of observations of the stated parameter; F, F-value of the ANOVA; p, *P*-value. Green shading indicates significant *P*-value (< 0.05); faint green shading indicates near-significant *P*-value (0.05 – 0.06).

**Supplementary Table 1c: Behavioural pharmacology, between-subject data analysed with univariate ANOVA and subsequent pair-wise ANOVA at each dose.** LMA, locomotor-activity; Fig., figure panel containing the analysed data; Ctrl, littermate wildtype controls, KO, CD2-KO mice; df, degrees of freedom in the ANOVA; n, number of animals in the indicated group; F, F-value of the ANOVA; p, *P*-value. Green shading indicates significant *P*-value (< 0.05).

**Supplementary Table 1d: Behavioural pharmacology, within-subject data analysed with repeated-measures ANOVA and subsequent pair-wise univariate ANOVA at each dose.** LMA, locomotor-activity; Fig., figure panel containing the analysed data; Ctrl, littermate wildtype controls, KO, CD2-KO mice; df, degrees of freedom in the ANOVA; n, number of animals in the indicated group; F, F-value of the ANOVA; p, *P*-value. Green shading indicates significant *P*-value (< 0.05).

| **Average age at start of experiment** |  | **Age (weeks)** | |
| --- | --- | --- | --- |
| **Behavioural test** | **Figure** | **females** | **males** |
| Novelty-induced locomotion | 1b-c, 5a | 12 | 15 |
| Amphetamine-induced locomotion | 1d-f | 43 | 46 |
| Locomotion: raclopride | 1h-j | 37 | 39 |
| Locomotion: SCH23390 | 1l-n | 56 | 65 |
| Locomotion: LY379268 | 1p-r | 41 | 45 |
| Locomotion: Clozapine | S1b-d | 62 | 68 |
| Spatial reference learning | 2b-c | - | 15 |
| Spatial rule-shift | 2e-f | - | 16 |
| Spatial rule-reversal | 2h-i | - | 18 |
| Operant rule-shift | 2k-l | - | 38 |
| Spatial novelty preference | 3b-f | 12 | 13 |
| Novel-object-recognition | 3h-k | 42 | 65 |
| 5CSRTT - training & baseline | 3m-p | 21 | 24 |
| 5CSRTT - challenges | 3q-t | - | 32 |
| T-maze training & challenges | 4b-e | 33 | 43 |
| T-maze: LY379268 | 4f | 59 | 55 |
| Elevated Plus-Maze | 5c-d | 13 | 14 |
| Sucrose preference & nest building | 5f-j | 47 | 52 |
| Social interaction | 5l-n | 36 | 41 |

**Supplementary Table 2: Age of mice at each experiment.** For each behavioural test, the average age of the mice at the beginning of the test is stated in weeks.

**Supplementary Table 3a: Within-subject data on cognitive flexibility analysed with repeated-measures ANOVA.** Fig., figure panel containing the analysed data; Ctrl, littermate wildtype controls, KO, CD2-KO mice; df, degrees of freedom in the ANOVA; n, number of animals in the indicated group; F, F-value of the ANOVA; p, *P*-value. Green shading indicates significant *P*-value (< 0.05); faint green shading indicates *P*-values < 0.05 reached in the Greenhouse-Geiser measure of the repeated-measures ANOVA. ***** Analysis includes the 2 knockouts and 1 wildtype mouse that did not reach criterion within 23 sessions and corresponds to the interval-data displayed in Figure 2k and Supplementary Figure 2. The row above corresponds to the analysis of the same dataset, albeit with those 3 mice excluded – this dataset is therefore identical to the cohort for which the sessions-to-criterion measure is displayed in main Figure 2l. Both ways of analysis yield identical results regarding significant effects of genotype. ** For all within-subject analysis, the conservative lower-bound measure from the repeated-measures ANOVA is stated. Therefore the degrees of freedom are identical for the within- and the between-subject analysis of a given dataset. #correct unlit, number of correct responses into a hole which was not illuminated; #correct lit, number of correct responses into a hole which was also illuminated; #incorrect unlit, number of incorrect responses in trials where the correct hole was not illuminated; #incorrect lit, number of incorrect responses in trials where the correct hole was also illuminated.

**Supplementary Table 3b: Between-subject data on cognitive flexibility analysed with *t*-test.** Fig., figure panel containing the analysed data; Ctrl, littermate wildtype controls, KO, CD2-KO mice; df, degrees of freedom in the *t*-test; n, number of animals in the indicated group; F, F-value of the ANOVA; p, *P*-value. Purple shading indicates adjustments of df and *P*-value due to unequal variances. Green shading indicates significant *P*-value (< 0.05). faint green shading indicates near-significant *P*-value (0.05 – 0.06). #incorrect dark, responses into holes 1, 3 and 5 which were never illuminated nor reinforced in any training stage (random errors).

# Supplementary References

1 Sicinski P, Donaher JL, Geng Y, Parker SB, Gardner H, Park MY *et al.* Cyclin D2 is an FSH-responsive gene involved in gonadal cell proliferation and oncogenesis. *Nature* 1996; **384**: 470–474.

2 Bygrave AM, Masiulis S, Nicholson E, Berkemann M, Barkus C, Sprengel R *et al.* Knockout of NMDA-receptors from parvalbumin interneurons sensitizes to schizophrenia-related deficits induced by MK-801. *Transl Psychiatry* 2016; **6**: e778.

3 Imre G. The Preclinical Properties of a Novel Group II Metabotropic Glutamate Receptor Agonist LY379268. *CNS Drug Rev* 2007; **13**: 444–464.

4 Young JW, Kooistra K, Geyer MA. Dopamine Receptor Mediation of the Exploratory/Hyperactivity Effects of Modafinil. *Neuropsychopharmacology* 2011; **36**: 1385–1396.

5 Lapin IP, Rogawski MA. Effects of D1 and D2 dopamine receptor antagonists and catecholamine depleting agents on the locomotor stimulation induced by dizocilpine in mice. *Behav Brain Res* 1995; **70**: 145–151.

6 Bygrave AM, Masiulis S, Nicholson E, Berkemann M, Sprengel R, Harrison P *et al.* Knockout of NMDA-receptors from parvalbumin interneurons sensitizes to schizophrenia-related deficits induced by MK-801. *Transl Psychiatry* 2016.

7 Sanderson DJ, Good MA, Skelton K, Sprengel R, Seeburg PH, Rawlins JNP *et al.* Enhanced long-term and impaired short-term spatial memory in GluA1 AMPA receptor subunit knockout mice: Evidence for a dual-process memory model. *Learn Mem* 2009; **16**: 379–386.

8 Sanderson DJ, Hindley E, Smeaton E, Denny N, Taylor A, Barkus C *et al.* Deletion of the GluA1 AMPA receptor subunit impairs recency-dependent object recognition memory. *Learn Mem* 2011; **18**: 181–190.

9 Bari A, Dalley JW, Robbins TW. The application of the 5-choice serial reaction time task for the assessment of visual attentional processes and impulse control in rats. *Nat Protoc* 2008; **3**: 759–767.

10 Papaleo F, Erickson L, Liu G, Chen J, Weinberger DR. Effects of sex and COMT genotype on environmentally modulated cognitive control in mice. *Proc Natl Acad Sci* 2012; **109**: 20160–20165.

11 Deacon RMJ, Rawlins JNP. T-maze alternation in the rodent. *Nat Protoc* 2006; **1**: 7–12.

12 Carlen M, Meletis K, Siegle JH, Cardin JA, Futai K, Vierling-Claassen D *et al.* A critical role for NMDA receptors in parvalbumin interneurons for gamma rhythm induction and behavior. *Mol Psychiatry* 2012; **17**: 537–548.

13 Deacon RM. Assessing nest building in mice. *Nat Protoc* 2006; **1**: 1117–1119.
